# Influences of the circadian clock on neuronal susceptibility to excitotoxicity

**DOI:** 10.3389/fphys.2013.00313

**Published:** 2013-11-05

**Authors:** Sumedha W. Karmarkar, Shelley A. Tischkau

**Affiliations:** Department of Pharmacology, Southern Illinois University School of MedicineSpringfield, IL, USA

**Keywords:** circadian rhythms, neurodegeneration, excitotoxicity, suprachiasmatic nucleus, MAPK

## Abstract

Stroke is the third leading cause of death and the primary cause of morbidity in the United States, thus posing an enormous burden on the healthcare system. The factors that determine the risk of an individual toward precipitation of an ischemic event possess a strong circadian component as does the ischemic event itself. This predictability provided a window of opportunity toward the development of chronopharmaceuticals which provided much better clinical outcomes. Experiments from our lab showed for the first time that neuronal susceptibility to ischemic events follows a circadian pattern; hippocampal neurons being most susceptible to an ischemic insult occurring during peak activity in a rodent model of global cerebral ischemia. We also demonstrated that the SCN2.2 cells (like their *in vivo* counterpart) are resistant to excitotoxicity by glutamate and that this was dependent on activation of ERK signaling. We are currently working on elucidating the complete neuroprotective pathway that provides a barricade against glutamate toxicity in the SCN2.2 cells. Our future experiments will be engaged in hijacking the neuroprotective mechanism in the SCN2.2 cells and applying it to glutamate-susceptible entities in an effort to prevent their death in the presence of excitotoxicity. Despite the advancement in chronopharmaceuticals, optimal clinical outcome with minimal adverse events are difficult to come by at an affordable price. Superior treatment options require a better understanding of molecular mechanisms that define the disease, including the role of the circadian clock.

## Circadian rhythmicity

Perhaps the most ubiquitous and persistent environmental factor present throughout the evolution of modern species is the revolution of the earth about its own axis, creating a 24 h solar day. The consequent recurrent pattern of light and darkness endows a sense of time to organisms that live on this planet. The importance of this sense of time is accentuated by an internal clock that functions on a 24 h scale, inherent in the genetic framework of living organisms ranging from cyanobacteria (Johnson et al., 1996) to human mammals (Herzog and Tosini, 2001). An internal, molecular program drives circadian oscillations within the organism that manifest at the molecular, biochemical, physiological and behavioral levels (Mazzoccoli et al., 2012). Importantly, these oscillations allow anticipatory responses to changes in the environment and promote survival.

The term “circadian” comes from the Latin “circa,” meaning “around” and “diem,” meaning “day.” Circadian events recur during the subjective day or the lighted portion of the 24 h period and the subjective night or the dark part of the 24 h period allowing physiological synchrony with the light/dark environment (Reddy and O'Neill, 2010). The circadian clock has been demonstrated in almost all living organisms (Johnson et al., 1996; Herzog and Tosini, 2001; Mazzoccoli et al., 2012). The two defining characteristics of the circadian timing system are perseverance of oscillation under constant environmental conditions, which define these rhythms as self-sustained and endogenously generated, and the ability to adapt to environmental change, particularly to changes in the environmental light/dark cycle (Tischkau and Gillette, 2005). Endogenous rhythms can be entrained to the outside world by several external cues of which light is the most widely studied and probably the best understood. Such external cues are called as “zeitgebers” or “time-givers” (Tischkau et al., 1999). Although other “zeitgebers” such as seasonal changes and daily temperature variations can also entrain internal rhythms to the outside world, they are currently outside the purview of this article and will not be discussed any further. Rhythms have been studied in whole animals (Tischkau et al., 2000), individual tissues (Tischkau et al., 2000), organs (Xu et al., 2010; Tischkau et al., 2011; Wang et al., 2011) and cell culture models (Welsh et al., 1995; Allen and Earnest, 2002). Perhaps the most obvious of all circadian rhythms is the behavioral rest/activity cycle (Ibuka et al., 1977; Mouret et al., 1978), which has been the subject of intense study, providing a behavioral standard for investigation of rhythmicity. Circadian cycles are also expressed in physiology and gene expression (Barnes et al., 2003; Karman and Tischkau, 2006).

Coherent circadian rhythms in mammals may be viewed as an organismic network. In the complex system of mammals, the master circadian pacemaker is the SCN (suprachiasmatic nucleus), located in the hypothalamus, above the optic chiasm from which it derives its name. Although individual neurons within the SCN are independent oscillators, organismic rhythm generation is considered an emergent property of the nucleus (Tischkau and Gillette, 2005). Nestled in the optic chiasm, and connected to the external environment through a direct, monosynaptic connection from specialized retinal ganglion cells, the SCN monitors environmental levels of illumination, which are integrated and information based upon these data transmitted to the rest of the organism through a variety of neuroendocrine mechanisms (Moore, 1993; Hannibal, 2002; Fabbian et al., 2013). Thus, the SCN harnesses and coordinates individual biological clocks located in several different tissues and organs in the body and thus is able to control multiple physiological processes and maintain their cyclical pattern in synchrony with the outside world. So, what controls the master clock? The answer lies in a molecular feedback loop consisting of the core group of clock genes (Lincoln et al., 2003).

The core molecular clockworks consists of several major genes including Per (Period), Tim (Timeless), Cry (Cryptochrome), Clock (Circadian Locomotor Output Cycles Kaput) and Bmal1 (Brain Muscle ARNT-Like 1; ARNT = Aryl hydrocarbon Receptor Nuclear Translocator) (Lincoln et al., 2003). Molecular interactions between these genes govern the central time-keeping mechanism in the suprachiasmatic nucleus neurons and help to synchronize them in order to maintain the clock integrity (Siepka et al., 2007). Typically, intracellular levels of Clock remain constant throughout the 24 h period. BMAL1 expression is high during the day and low at night. When present in sufficient levels, BMAL1 heterodimerizes with CLOCK and together they bind to E-box sequences in the promoter region of Cry (Cry1, 2) and Per (Per1, 2, 3) genes. The BMAL1-CLOCK heterodimer also turns off Bmal1 transcription. The results of BMAL1-CLOCK activity are, therefore, reduction of Bmal1 transcript levels and concomitant activation of Per and Cry at the beginning of the day. Similar to BMAL1-CLOCK, Per and Cry form heterodimers that enter the nucleus at the beginning of night. CRY disrupts the CLOCK-BMAL1 interaction at E-box elements. As a result, BMAL1 levels drop, thus reducing the amount of the BMAL1-CLOCK heterodimer, thereby suppressing transcription of Per and Cry, and allowing transcription of BMAL1. Regulation of the BMAL1-CLOCK complex by ancillary feedback loops provides stability to the molecular oscillator. The nuclear receptors ROR1α (Retinoid-related Orphan Receptor 1α) and Rev-erbα provide positive and negative regulation of the BMAL1 promoter, respectively. The interacting positive and negative feedback loops of circadian genes ensures low levels of Per and Cry, and a high level of Bmal1 at the beginning of a new circadian day (Siepka et al., 2007). In Drosophila, the Tim gene interacts with Per to act as a negative regulator of the CLOCK and the fly homolog of BMAL1, CYCLE. (Bae et al., 1998). Disruption of any one of these components results in a dysfunctional clock and an eventual state of less than optimum physiology (Ohdo, 2010; Doi, 2012).

## The circadian clock and disease

Decades of research has time and again demonstrated the importance of the internal time-keeping mechanism in maintaining optimum integrity of the organism as a whole. The SCN controls several core physiological processes which include, but are not limited to, heart rate (Morris et al., 2012), core body temperature (Li et al., 2012), glucose metabolism (Li et al., 2012), cortisol levels (involved in controlling blood pressure) (Clow et al., 2010), sleep-wake cycle (Franken and Dijk, 2009) and hormones of the reproductive cycle (Swaab et al., 1996). Surgical or electrolytic lesioning of the suprachiasmatic nucleus results in a state of complete arrhythmia in rodents (Mosko and Moore, 1979). Thus, the central molecular clock coordinates the activities of clocks residing in peripheral tissues and in this way entrain several biological process to the outside world (Brown and Azzi, 2013). Alteration of the clock and/or expression or suppression of one or more clock genes, cause deviation from homeostasis in the physiology of an organism and is predictive of disease (Mazzoccoli et al., 2012). Such altered patterns are quite typically observed in the livers of animals subjected to a high fat diet (Shi et al., 2013), or humans undergoing shiftwork (Berger and Hobbs, 2006) and jetlagged animals (Filipski et al., 2004). Recent studies have demonstrated a strong correlation between a disrupted clock and an increased incidence of several diseases and disorders such as obesity (Shi et al., 2013), metabolic syndrome (Xu et al., 2010), diabetes (Wang et al., 2011), breast cancer (Haus and Smolensky, 2013), sleep disorders (Haus and Smolensky, 2013), depression (Li et al., 2013) and certain neurological conditions (Jagannath et al., 2013). In order to demonstrate the tremendous importance of clock genes in physiology, we have incorporated a brief list of clock gene malfunction, matched to the corresponding pathological state that its malfunction manifests, as summarized in Table 1 below. We are of course aware of the tremendous proportions of literature available that barely even begins to describe the ramifications of circadian disruption in pathology of several diseases and disorders. We are not providing a comprehensive list, but just a brief glimpse of the role of clock gene malfunction in mediating, maintaining or exacerbating pathology.

**Table 1:** **Clock gene malfunction and associated pathology**.

**Clock gene malfunction**	**Manifested pathology**
Clock-mutant	Metabolic syndrome (Turek et al., 2005)
CLOCK and BMAL1 disruption	Hypoinsulinaemia and diabetes (Marcheva et al., 2010)
Cry-deficiency	Hyperinsulinemia and tissue-specific insulin resistance (Barclay et al., 2013)
Per-deficiency	Increased spontaneous and radiation-induced tumor development (Lee et al., 2010)

Excitingly, a picture is gradually emerging that progression and fates of many diseases are tied to the health of the clock; a healthy body contains a healthy clock. Disruption of clock function may be an important contributor in disease. Several studies demonstrate the importance of a healthy clock to cardiovascular health. One study used transverse aortic constriction to induce compensated heart and valve disease (characterized by cardiac hypertrophy and hyperplasia) in mice (Martino et al., 2007). This condition was exacerbated in mice subjected to a lighting schedule that differed from their regular day-night pattern. Cardiac clock gene profiling revealed a gross alteration in the rhythmic gene expression. The pathological state of the cardiac tissue was rescued after the rodents were reverted to their regular day-night cycling (Martino et al., 2007). A similar study demonstrated the importance of the biological clock in maintaining normal physiology. Hamsters with the tau mutation have severe cardiac and renal morbidities and a shortened lifespan (Martino et al., 2008). This is because the tau mutation results in a shorter period and therefore these animals cannot adapt to the 24-hour cycle. However, when placed in a cycle that is normal for this mutation i.e., the 22-h cycle, the hamsters display normal cyclical behaviors and a normal cardio-renal phenotype characteristic of a healthy animal (Martino et al., 2008). Other studies have utilized clock gene knockout mice for demonstrating the importance of a healthy circadian rhythm in maintaining normal cardiovascular physiology. One such study conducted, involved the induction of cardiovascular pathology in Bmal1 knockout mice and Clock mutant mice by common carotid artery ligation (Anea et al., 2009). Ligation of the common carotid artery mimics the pathological course of cardiovascular diseases such as atherosclerosis and hypertension that occurs in human beings. This study showed that common carotid artery ligation triggers a dramatic cardiac remodeling response that attempts to salvage cardiac tissue from forming scar tissue and rescues optimal function. However, in mice with a disrupted circadian physiology (Bmal1 knockout and Clock mutant mice), common carotid artery ligation prompts a less than optimum response, which results in a worsening cardiovascular pathology as compared to their wild-type counterparts (Anea et al., 2009). Whether clock disruption is the cause or the outcome of the diseased conditions remains largely unknown; but, partial restoration of clock function appears to alleviate the symptoms of such diseases, slow down the progression or improve the outcomes of treatments (Sewlall et al., 2010; Hermida et al., 2013).

Considering that cardiovascular diseases rank first in terms of long-term treatment and mortality, it is not surprising that these conditions warrant the utmost urgency in determining therapeutic strategies for reducing the tremendous burden on the healthcare system. The cardiovascular system has long since been known to harbor circadian components (Paschos and FitzGerald, 2010). It is common knowledge that certain aspects of the cardiovascular system vary with time of day, such as sympathetic tone, vascular reactivity, heart rate and blood pressure (Paschos and FitzGerald, 2010). Ramifications of these circadian events extend to factors that govern the onset, maintenance and progression of cardiovascular dysfunction including stroke (Uen et al., 2009). Observational studies and a recent meta-analysis have shown that stroke and cardiac arrest occur predominantly in the morning (Hassler and Burnier, 2005; White, 2007; Patel et al., 2008; Uen et al., 2009), thus demonstrating that just as the physiological aspect of the cardiovascular system is governed by a circadian overlord, so is its dysfunctional state governed by a time-of-day variation.

## Circadian rhythms and stroke

Stroke is the third leading cause of death and the primary cause of morbidity in the United States. Basic science and clinical research have repeatedly revealed that stroke, among other cardiovascular events such as myocardial infarction, sudden cardiac death and arrhythmias, has a propensity to precipitate at a particular time-of-day; wherein these events are most likely to occur during the hours of 6:00 a.m. and 12:00 p.m. (Hassler and Burnier, 2005; Manfredini et al., 2005; White, 2007; Patel et al., 2008; Uen et al., 2009). Not only is the incidence of an ischemic event (such as acute myocardial infarction) higher during the morning hours, but the mortality resulting from such an event was also higher during this time (Manfredini et al., 2004). A retrospective clinical human study, demonstrated that myocardial injury and decreased LV (Left Ventricular) function was the worst when STEMI (ST-Elevation Myocardial Infarction) occurred in the early morning hours and when reperfusion followed 4 h later (Reiter et al., 2012). This timeline is reminiscent of the sleep-wake transition period. A similar study assessed the effect of time-of-day with respect to myocardial injury occurring in STEMI patients undergoing PPCI (Primary Percutaneous Coronary Intervention) (Fournier et al., 2012). When STEMI occurred in the early morning hours (between 00:00 and 05:59) infarct size was greater, with increased damage to myocardial tissue and a higher mortality at 30-days. This study therefore concluded that time-of-day should be factored in when assessing the prognosis after STEMI (Fournier et al., 2012).

Primary evidence of this early morning susceptibility has been linked to a variety of factors that are activated or inhibited during this time, many of which possess a strong circadian component. Although causative data to evaluate mechanisms underlying these diurnal variations are lacking, circadian rhythms in activity, blood pressure, platelet aggregatability, cortisol and catecholamines are candidate factors (Bridges and Woods, 2001; Hassler and Burnier, 2005; White, 2007; Patel et al., 2008; Uen et al., 2009). These factors contribute to greater blood coagulability during the early morning hours, rendering the subject more susceptible to the adverse effects of ischemic events. Other factors that might also contribute to a greater coagulable state are plasma cortisol, plasma renin activity, angiotensin II, aldosterone, arterial stiffness, vascular resistance, blood viscosity, (Hassler and Burnier, 2005) as well as “increased reactive oxygen species formation by mononuclear cells, increased ubiquitin-proteasome activity (associated with inflammation-induced plaque rupture) in atherosclerotic lesions, greater common carotid artery intima-media thickness values, physical exertion, emotional state (anger) and sexual activity” (Patel et al., 2008). Any one of the above mentioned factors or a combination of some might trigger an ischemic event resulting in a stroke.

Data collected over the past decade suggest a causative link between hypertension, commonly referred to as a “silent killer” and stroke (Dinsdale et al., 1978). Among all the factors that contribute to a stroke, blood pressure is the one factor which has displayed a very strong correlation to stroke incidence and clinically is the most practical to measure and manipulate. Some studies demonstrated that maintaining blood pressure within normal limits during the peak susceptibility hours (early morning) can reduce adverse cardiovascular events, stroke in particular (White, 2007; Patel et al., 2008). On the other hand, some other researchers alleged that it was the night-time blood pressure that predicated the risk of an individual toward a stroke (Uen et al., 2009; Yano and Kario, 2012a,b). These studies have used a classification system for determining the population at highest risk for stroke. This system is based on the “dipping status” of an individual. These studies classified subjects as “dippers” (blood pressure dropped at night), “non-dippers” (blood pressure did not drop at night) and “inverted dippers” or “risers” (blood pressure was increased at night). These studies demonstrated that the “non-dippers” and “risers” were more susceptible to ischemic events and that controlling the rise of blood pressure at night would produce a better outcome than that obtained by controlling the early morning surge. In either case, all studies provided irrefutable evidence that ischemic events follow a certain circadian pattern. Irrespective of the cause of the ischemic event, this collective literature clearly showed that the factors contributing to an ischemic event follow a circadian pattern that corresponds with the actual event.

Neuronal susceptibility to death increases with age and several factors come into play, which determine how an individual's body will react to a certain ischemic event: environmental factors such as diet, exercise, individual habits (drinking, smoking, chewing tobacco, addiction to drugs), stress, presence of co-morbidities (obesity, diabetes), family history, genetic tendencies and of course, age. A vast amount of empirical evidence suggests that with progression in age, several aspects of the physiology become less efficient with time, and the time-keeping system is no exception. Tested by time, classic circadian studies have demonstrated that the aging process interferes with the time-keeping system (Weitzman et al., 1982; Czeisler et al., 1999; Duffy and Czeisler, 2002). Our findings clearly show that pathological conditions are associated with deviation of the clockwork system from its original, physiological state (Tischkau et al., 2007).

A more recent and sophisticated approach toward studying factors that put certain individuals at a greater risk for a certain event than the general population, is the use of genetic biomarkers. This approach is gaining rapid popularity due to the availability of advanced instrumentation and the cardiovascular field is no exception. Although blood pressure management provided a great therapeutic outcome in the treatment of several cardiovascular diseases in the past, it was by no means without several disadvantages. Blood pressure has a very strong circadian component (Bridges and Woods, 2001) and that can act as a double-edged sword; while it provides a good means for identifying individuals at a high risk for adverse cardiovascular events and can be practically manipulated, it is usually measured during an office visit by the patient (White, 2007). Round-the-clock portable measuring cuffs provided some resolution to that problem (White, 2007), but, it is rather impractical to ask a patient to wake up every other hour to take an accurate reading of their blood pressure and take their medication accordingly. Hence, there is a tremendous need to find better treatment approaches and the answer lies in first gaining a better understanding of the disease itself.

## Diurnal variation in neuronal damage incurred by ischemic insult in a rat model

Several physiological factors such as inherent increased coagulability in the early morning or the presence of comorbidities such as hypertension and cardiovascular diseases predispose subjects to an ischemic event (Patel et al., 2008). However, none of the studies thus far have ventured to determine whether an inherent underlying susceptibility to neuronal damage at a particular time of day would exacerbate the extent of damage caused by an ischemic event as opposed to an identical event occurring at a time of day when the inherent neuronal susceptibility to damage was lower. In a clinical setting, it is difficult to establish such a cause and effect relationship. Yet studies using animal models to predict susceptibility and damage to ischemic insult are sparse. In one study, rats were subjected to middle cerebral artery occlusion at various times of the day (Tischkau et al., 2007). Rats were most sensitive to the effects of ischemia at the beginning of their active period and least susceptible at the beginning of their inactive period (Tischkau et al., 2007). Infarct volume was more than three times greater during the most sensitive time of day than it was during the insensitive period. Based on this information, a rat model was developed to evaluate the effect of time of day on the extent of damage induced by an ischemic event in the hippocampus; a region of the brain that is highly susceptible to stroke in humans.

Rats were maintained in a 12-12 LD (light-dark) cycle, with the day beginning at ZT0 (Zeitgeber time 0, defined as the time of lights “on” at 7:00 a.m.) and ending at ZT12 (lights “off” at 7:00 p.m.). The dark period spanned from ZT12 to ZT24/0. Surgically induced cardiac arrest produced a global ischemia that resulted in reproducible and consistent episodes of ischemic events. A thoracotomy was performed under isoflurane anesthesia; the aortic root and pulmonary artery were clamped for 4 min, under conditions of no ventilation resulting in cardiac arrest and inhibition of total blood flow to the entire brain. Animals were kept on heating pads during the entire procedure to nullify the neuroprotective effects of hypothermia. The study addressed 2 major questions: (1) Does time-of-day at which ischemic insult occur affect the expression of biomarkers preceding cell death in the hippocampus? (2) Does an ischemic insult cause a deviation in the expression pattern of clock genes from that of the normal pattern of expression? The hippocampus was the brain region under investigation because this region is particularly susceptible to damage during the aftermath of a stroke. In order to assess the damage occurring after an ischemic event, several cell death markers such as caspase 3, caspase 8 and caspase 9 were employed. Programmed cell death, or apoptosis, is an evolutionarily conserved process that plays a major role in development and in maintenance of tissue homoeostasis (Cohen, 1993). Furthermore, apoptosis is a common feature of many neurodegenerative disorders (Rubin et al., 1994), including stroke (Choi, 1996). Thus, apoptosis is an inherent part in health and disease. Apoptotic processes are classified into 2 major pathways: 1) the intrinsic (or mitochondrial pathway, involving caspases 3 and 9 and 2) extrinsic (or death receptor pathway, involving caspase 8 pathways (Debatin, 2004). Activated caspases 3, 8, and 9 have been used as reliable markers for predicting cell death as an outcome in a vast array of studies ranging from *in vitro* cell culture to *in vivo* animal models, in different types of tissues such as cardiac tissue and brain tissue and in varying operator hands in labs all over the world.

Animals were subjected to global ischemia by cardiac arrest at ZT6, ZT14, and ZT20 and sacrificed 24 h later (Tischkau et al., 2007). These time-points were chosen because ZT6 is a time-point of peak rest period and ZT14 and ZT20 are time-points of activity-onset and late activity periods respectively. ZT6 is analogous to activity-onset in humans whereas ZT14 and ZT20 would be periods much later than the activity-onset time-point. This difference stems from the fact that while rodents are nocturnal (peak activity during the dark period), humans are diurnal (peak activity during the light period). A significant elevation of caspase-3 mRNA was observed in ischemic hippocampi at ZT14 (early in the active period). Also, although there was a small but significant increase in the protein levels at ZT6, a dramatic increase in caspase-3 protein levels was evident when the ischemic insult was initiated at ZT14. Similarly, although there was a significant increase in caspase-8 mRNA at ZT6 (2.9 fold), the increase at ZT14 was much greater (10.0 fold). However, the protein levels were significantly elevated only with respect to the sham controls; time-of-day did not seem to play a part in the extent of elevation of caspase-8 protein levels. Caspase-9 transcript levels were increased at ZT6 (3.3 fold) and ZT20 (3.3 fold); however, the increase at ZT14 was much higher (8.3 fold). Following the same trend, caspase-9 protein levels after ischemia were substantially higher at ZT14 (15 fold) than at ZT20 (2.4 fold); there was no change in the caspase-9 protein levels at ZT6. It is also important to note here that the transcript and protein levels for caspases 3, 8 and 9 did not change in the sham controls with respect to time of day. These results strongly suggest that damage occurring in the aftermath of an ischemic insult initiated in the early night (ZT14) is drastically greater than the damage occurring after an insult initiated at a different time in the nocturnal cycle.

One of the popular theories explaining neuronal death is the “calcium overload theory” (Lok and Martin, 2002). According to this theory, an excitotoxic insult such as excessive glutamate release, results in extensive depolarization of a neuron, which prompts the **N-methyl D-aspartate Receptors (NMDARs)** to open. These channels promote a massive calcium deluge into the cell. Persistent activation of these channels causes a “calcium overload” into the neuron which the cell cannot get rid off fast enough. This causes over-activation of all calcium-dependent processes, which provides conflicting information on what cell process to activate, deactivate or maintain. Subsequently, unable to keep up with the arrival of conflicting messages, the system goes into over-drive and exhausts itself, killing the cell.

The physiological defense system against this calcium overload is executed by a family of calcium buffering proteins such as calretinin, parvalbumin and calbindin. Several studies have time and again demonstrated the importance of calbindin in buffering the excess calcium entering a neuronal cell during an ischemic excitotoxic insult. Calbindin overexpression protected striatal neurons from the aftermath of transient focal cerebral ischemia (Yenari et al., 2001). A couple of other studies have also shown that calbindin-expressing neurons are better equipped to survive ischemic and excitotoxic insults (Robier and Le Scao, 1991). Two other studies also clearly demonstrated the benefits of short-term overexpression of calbindin in protecting neurons from an excitotoxic insult *in vitro* and *in vivo* (Yenari et al., 2001; Fan et al., 2007). Therefore, expression of calbindin, or lack thereof might determine propensity toward exacerbated damage in presence of an excitotoxic insult such as stroke. In the cardiac arrest model, calbindin levels were significantly reduced at all time-points, but the greatest reduction was observed at ZT14. This taken together with the caspase data further strengthens the idea that regions of the brain susceptible to excitotoxic insult are inherently more susceptible at specific times-of-day. Inherent neuronal susceptibility to this ischemic event follows a circadian pattern.

To further elaborate on the circadian nature of inherent neuronal susceptibility to ischemic damage, changes in the pattern of expression of Clock genes were determined. The typical expression pattern is indicative of a healthy clock (Reddy and O'Neill, 2010). Any kind of deviation from the physiological expression of these genes suggests a perturbation in the normal physiology. Consistent with previous reports, in untreated controls, *Clk* expression did not change with respect to time of day while Per1 and Cry1 were in-phase with each other (peaking during the early morning and late night, with a trough during the later day and early night). Bmal1 expression was out-of-phase with that of Per1 and Cry1. Animals were subjected to global ischemia at ZT6 and hippocampi were collected at 0 (ZT6), 6 (ZT12), 12 (ZT18), 18 (ZT24), and 24 (ZT6, the next day) hours after surgery. The sham controls followed the same expression pattern as the untreated controls. The expression patterns of Bmal1 and Cry1 were unaffected by global ischemia. Per1 levels on the other hand were significantly affected by ischemia. Ischemia resulted in Per1 elevation at 6 h and 12 h after the ischemic insult when compared to the sham controls. The Per1 levels at 18 h and 24 h after ischemia were significantly lower than their sham-operated counterparts. Taken together, these results suggest that ischemia resulted in a phase-advance of Per1 expression. This study provided strong evidence for the existence of inherent susceptibility to neuronal damage that varies with time of day.

## The SCN and resistance to excitotoxicity

**Figure 1:**
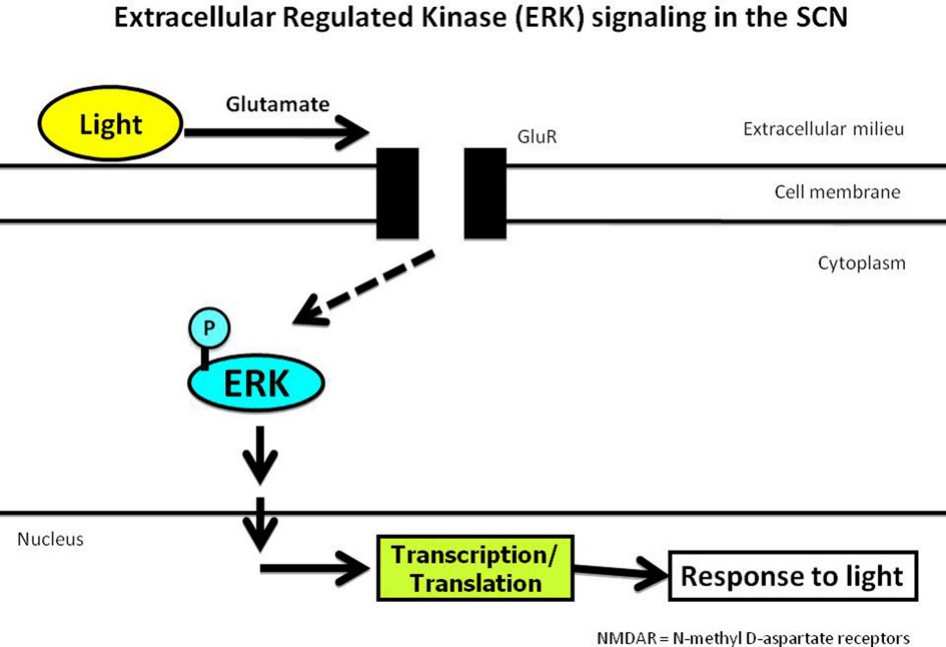
**Light-induced ERK phosphorylation is required for producing an appropriate response to the light stimulus**.

The previous study elaborately described the inherent susceptibility of neurons to ischemia that changes with respect to time-of-day, but made no attempt to explore mechanisms that may underlie the susceptibility to ischemic damage. Glutamate is the major excitatory neurotransmitter in the brain but its release is tightly regulated by a complex array of biochemical reactions (Westbrook, 1993). However, under several pathological conditions, excessive glutamate release results in severe neuronal obliteration (Michaelis, 1993). The hippocampus is highly susceptible to glutamate-induced damage and several theories suggest that glutamate may be a causative factor in the degeneration caused by stroke and other neurodegenerative diseases (Coyle and Puttfarcken, 1993). However, under physiological conditions certain populations of neurons are naturally resistant to glutamate insult; the SCN neurons are among those that are resistant. Figure 1 depicts the typical ERK (Extracellular Regulated Kinase) signaling in response to light in the SCN. Physiologically, light activates retinal ganglion cells that project onto the SCN and release glutamate (Kornhauser et al., 1996). Glutamate release results in activation of glutamate receptors (GluR) that in turn activate ERK (by phosphorylation). Activated ERK is required for several transcriptional and translational changes that are essential for turning the hands of the clock. Previous studies have demonstrated that blocking ERK inhibits the response to light (Butcher et al., 2003). This process of glutamate release occurs for as long as light continues to activate the retinal ganglion cells. As a result, the retino-recipient region of the SCN is subjected to a continuous glutamate barrage. Unlike the hippocampal neurons, SCN cells thrive in this seemingly “toxic” glutamate environment. Attempts to lesion the SCN using excitotoxins fail; the SCN continues regular functioning even after glutamate treatment. Histological evidence failed to demonstrate neuronal death in the SCN neurons after ischemia or excitotoxin treatment.

Mechanisms that underlie excitotoxic resistance in SCN neurons were explored in the SCN2.2 cell line (Bottum et al., 2010). The SCN2.2 cell line is derived and immortalized from the rat SCN (Earnest et al., 1999). Previous studies have reliably and reproducibly demonstrated that this cell line adheres to the features of the SCN *in vivo* i.e., its clock properties (Allen and Earnest, 2002). More importantly, the SCN2.2 cells are resistant to glutamate insult (Bottum et al., 2010). However, mechanisms that underlie the resistance of these peculiar neurons in the presence of glutamate remain under investigated. This was of special interest because these neurons are the major time-keepers in our brain i.e., they belong to the SCN. The GT1-7 cell line, derived from the GnRH (Gonadotropin Releasing Hormone) neurons from the mouse hypothalamus (Liposits et al., 1991), was used as a control. GT1-7 cells are susceptible to 5 mM glutamate whereas the SCN2.2 cells thrive comfortably in 10 mM of glutamate (Bottum et al., 2010). While SCN2.2 cells survive up to 100 h in 10 mM glutamate, GT1-7 cells have reduced metabolic activity as early as 8 hours and their live population starts declining by 40 h (Bottum et al., 2010). The significance of the GT1-7 cell line is that it is derived from neurons that lie in close proximity to the SCN *in vivo*, but display neither the circadian properties nor the resistance to glutamate, both of which are possessed by the SCN neurons.

The “resistance” of the SCN neurons was initially thought to be due to inability of these neurons to respond to glutamate i.e., lack of the NMDAR which is infamously notorious for its propensity toward initiating and propagating neuronal damage under pathological conditions (Frandsen and Schousboe, 1987). However, several studies have shown that the SCN *in vivo* possesses functional NMDARs. In order to entrain the various physiological and behavioral rhythms to the environment, the endogenous clock must be reset periodically and it does this primarily by using light from the environment as a cue. Light reaches the SCN via a direct retinal projection, the RHT (Retino-Hypothalamic Tract) (Mosko and Moore, 1979; Moore, 1993). Light falling on the retina activates the optic nerve to carry photic information via a specific subset of retinal ganglion cells in the RHT to the SCN (Ebling, 1996; Mintz et al., 1999). Optic nerve stimulation releases glutamate onto the SCN (Liou et al., 1986). In hypothalamic slices, glutamate and NMDA (N-methyl D-aspartate) mimic the effects of light (Ding et al., 1994). NMDAR antagonists block these effects (Colwell and Menaker, 1992; Colwell et al., 1992). These data provide conclusive evidence that NMDARs present on the SCN are necessary and sufficient for SCN function. NMDAR subunit transcripts and protein are expressed in SCN2.2 cells (Bottum et al., 2010). However, the functionality of these receptors in the SCN2.2 cells remains undetermined as of now.

Evidence of gargantuan proportions demonstrates that MAPK (Mitogen-Activated Protein Kinases) determine cell fate (among a myriad of other functions that are outside the scope of this review) in neurons and other cell systems (Park et al., 2008; O'Sullivan et al., 2009; Zhong et al., 2009). There are 3 main mammalian MAPK molecules, namely, ERK (de Lemos et al., 2010), p38 (Han et al., 1995), and SAPK/JNK (Stress-Activated Protein Kinase; Jun N-terminal Kinase) (Minden et al., 1994). Although controversial, ERK has been largely demonstrated as a pro-survival kinase whereas p38 and SAPK/JNK are more involved in a cell death-mediating effect. Each of these kinases at some point have been purported to play an opposite role in determining cell fate. These ambiguities in outcomes are probably due to different cell types, tissues, investigation systems (*in vivo*, *in vitro* or *ex vivo*), different operators and a host of other factors. The role of each of these kinases in the SCN2.2 cell line in the presence of an excitotoxic glutamate insult was explored. In each case, the phosphorylated form of the kinase indicates its active state. In our study (Karmarkar et al., 2011), we found that glutamate consistently increased the phosphorylated (activated) ERK (p-ERK) levels in SCN2.2 cells; there was no change in the GT1-7 cells. At 48 h post glutamate treatment, p-ERK levels were significantly lowered in GT1-7 cells but still elevated in the SCN2.2 samples. These data are consistent with a pro-survival role for ERK signaling in the SCN2.2 cells. There was no change in the phosphorylated (activated) p38 (p-p38) levels in the glutamate-treated SCN2.2 cells, but they were increased in the glutamate-treated GT1-7 cells at 12 h post-glutamate treatment. At 48 h, the p-p38 levels remained unchanged in the SCN2.2 samples and were only slightly (but significantly) reduced in the glutamate-treated GT1-7 cells. These data are consistent with a pro-death role for p38 in the GT1-7 cells. The p-SAPK/JNK levels remained unchanged with treatment in both the cell systems at 48 h of treatment and for the entire short-term treatment in the GT1-7 cells. Previous literature suggests that although prolonged stress is often deleterious to a system, an acute and low-profile stress can be neuroprotective as it alerts the system to activate signaling mechanisms that prevent further damage to the system. This particular model for the role of SAPK/JNK fit well with the transient SAPK/JNK activation in SCN2.2 cells in response to a glutamate stressor. Thus, both ERK and SAPK/JNK are most likely responsible for inciting the neuroprotective signaling cascade in response to the glutamate insult.

Pre-treatment of the SCN2.2 cells with an ERK inhibitor (PD98059) prior to glutamate or NMDA treatment significantly increased the susceptibility of SCN2.2 cells to excitotoxicity, as assessed by the live-dead assay, metabolic activity assay, caspase-3 activity assay and cleaved (activated) caspase-3 immunocytochemistry. This cell death was prevented by the presence of the NMDAR blocker MK-801 (irrespective of whether glutamate or NMDA was used in the presence of the ERK inhibitor). These data demonstrated that the glutamate-mediated toxicity (in the presence of the ERK inhibitor) is dependent on NMDAR signaling.

**Figure 2:**
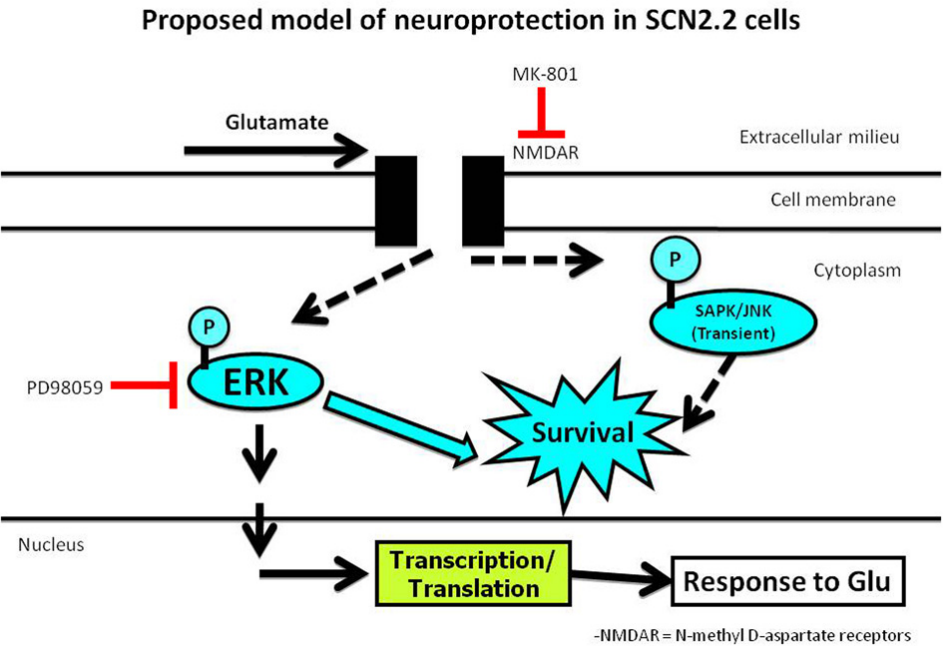
**ERK phosphorylation is essential for protecting the SCN2.2 cells from a glutamate insult**.

In summary, SCN2.2 cells, like their *in vivo* counterparts, are resistant to glutamate toxicity. The conclusions from our studies led us to propose a hypothetical model which is summarized in Figure 2 below. The SCN2.2 cells are damaged by glutamate if ERK is inhibited simultaneously. This glutamate-mediated toxicity is dependent on NMDAR signaling. However, ERK is a very “broad” kinase with myriad functions that vary with different systems under divergent conditions. Therefore, further studies should focus on determining the upstream regulators of ERK activation and narrowing down on the downstream effector molecules that prevent SCN2.2 cells from dying. Once a neuroprotective pathway has been established, the same pathway could be hijacked in neurons that are susceptible to glutamate damage and prevent their cell death in the presence of an excitotoxic insult. Furthermore, it is not just what signaling pathway should be hijacked but also what would be the appropriate time to initiate it and for how long it should be maintained in an activated state. The studies outlined here represent the first foray into understanding mechanisms that render certain neurons resistant to hostile excitotoxic conditions that would obliterate most other neurons in the brain. The investigation of the role of circadian properties in determining susceptibility or resistance to a certain cell fate is in its infancy. There is the need for tremendous research to be performed to seek out molecular targets which one day would result in successful therapies in the battle against the aftermath of ischemic events such as stroke.

Emerging evidence puts forward another possibility that time-keeping phenomenon inherent to the SCN is by itself a neuroprotective mechanism. Molecules such as melatonin and prokineticin that are responsible for allowing the SCN to keep time might themselves provide the SCN an inherent resistance to being “over-worked” i.e., protect against stressful conditions such as excessive glutamate. Melatonin is available over the counter as a remedy for insomnia, for fighting jet-lag, as an anti-oxidant (free radical scavenger) and an anti-inflammatory agent (Al-Omary, 2013). Several studies have time and again demonstrated that melatonin possesses neuroprotective properties in several neurodegenerative disorders (Carpentieri et al., 2012). Similarly, prokineticin serves as one of the chief output signals of the SCN (Li et al., 2012). Prokineticin-mediated activation of its receptor results in activation of G-coupled proteins which are in turn coupled with activation of MAPK/Akt (Ngan and Tam, 2008). By virtue of coupling of prokineticin to MAPK/Akt, prokineticin action mediates a broad spectrum of biological actions such as differentiation, proliferation and migration of target cells (Ngan and Tam, 2008). It is only a matter of time until the right pharmaceutical formulation is designed to safely administer melatonin and/or prokineticin and put their true potential to the test. Our current studies have been limited to using the SCN2.2 immortalized cell line. The major advantage of using a cell line is that several experiments can be performed simultaneously and are easily reproduced several times. However, the major drawback of this system is that it does not take into account the *in vivo* cyto-architecture and the intricate “network” of neuronal interactions. Subject to funding availability, we will continue to pursue similar studies, first in SCN slices, followed by whole animal studies *in vivo*.

## Chronopharmaceuticals and stroke

It is now largely accepted that a single office blood pressure measurement is not sufficient to determine whether an individual is essentially at a high risk for the precipitation of an ischemic event (Neutel and Smith, 1997). Therefore, the long-sustained practice of simply combining different anti-hypertensives for achieving better blood pressure management, no longer holds true. Portable blood pressure measurement devices helped give a better basis for sorting out those individuals who are at a high risk for an ischemic event (Neutel and Smith, 1997). Data from more recent studies have further strengthened the argument for incorporating ambulatory blood pressure monitoring as a part of standard care. This is particularly applicable to subjects who are at a greater risk of incidence of ischemic events. A recent article has described in exquisite the detail, the urgent need for ambulatory blood pressure management for the early diagnosis of asymptomatic, hypertensive subjects and as a clinical “marker” for assessing therapeutic outcome in patients already being treated for a myriad of post-ischemic diseases (International Society for et al., 2013). This would be especially pertinent in patients with concomitant disorders such as diabetes, chronic kidney disease, metabolic syndrome and sleep disorders, as these conditions could potentially adversely affect the therapeutic treatment of the ischemic event. However, continuous therapeutic monitoring being cost-prohibitive and repeated dosing at certain intervals being impractical provoked the need for a completely different treatment strategy. This is where chronopharmaceuticals are helping make headway into a practical therapeutic approach which produces optimal clinical outcome with least possible adverse events and is also financially affordable.

With the identification of the importance of circadian biology in health and disease (Reddy and O'Neill, 2010), considerable investments are ensuing to develop particular treatment regimens that focus on the importance of chronobiology; time-of-day is now being considered for its relevance to treatment efficacy with reduction in adverse reactions. This means that round-the-clock therapeutic clinical monitoring becomes essential in order to tailor the regimen according to the need of each individual subject. Although feasible in theory, executing this type of treatment strategy in practice is cost-prohibitive. The current efforts to overcome this hurdle lie in the development of chronopharmaceuticals.

Chronopharmaceuticals are drugs that are given at a particular time of day, predicated by the expected onset of an event at a particular time (Sewlall et al., 2010). One review article elegantly summarized several cardiovascular medications (beta-blockers, calcium channel blockers, nitrates and aspirin) that have been tested for their potential as a chronopharmaceutical for optimal efficacy (Bridges and Woods, 2001). Some of these drugs are already approved by the FDA and some are under investigation for better pharmacokinetics (including, but not limited to extended release formulations) (Sewlall et al., 2010). Several clinical trials are already in place to test chronopharmaceuticals in the prevention or mitigation of several diseases such as asthma, allergic rhinitis, arthritis, cancer and cardiovascular disorders including those precipitated by ischemic events (Sewlall et al., 2010). Past clinical trials involving timed dosing of individual anti-hypertensives have yielded mixed conclusions (Bridges and Woods, 2001). The current trend is toward exploring the efficacy of newer formulation types using drugs that are already approved by the FDA. Clinical trials using novel drug delivery systems have provided promising results; some of the most notable ones are the COER (Controlled-Onset, Extended-Release) −24 verapamil clinical trial, CODAS (Chronotherapeutic Oral Drug Absorption System) verapamil clinical trial, diltiazem (Cardizem XL) clinical trial and the CONVINCE (Controlled Onset Verapamil INvestigation of Cardiovascular Endpoints) clinical trial (White and LaRocca, 2002).

Although there are some controversies over whether controlling increased blood pressure at night or inhibiting the early-morning surge would produce better results, the undeniable conclusion attained at the end of these trials was that circadian events could be potentially harnessed for more effective treatment regimens resulting in optimal management of the disease with least possible adverse events. Thus, understanding the physiological factors that increase susceptibility to an ischemic event and more importantly, predicting when these factors are more likely to trigger such an event would provide superior management of a disease.

## Conclusions

Cardiovascular diseases rank the highest in terms of mortality. The risk for development of these diseases is based on a whole host of factors which essentially determine the state of coagulability of an individual; greater the coagulation factors present, the greater the incidence for precipitation of an ischemic event (White and LaRocca, 2002). The most practical to manipulate among these factors is blood pressure because it is non-invasive and can be performed by the subject outside the clinic (Pickering et al., 1985). Although portable blood pressure monitoring has become easy to report, the simple combination of a variety of anti-hypertensives has failed to produce the optimal results that it had promised.

Blood pressure has a strong circadian rhythm wherein it peaks upon onset of activity and dips during the period of least activity (Bridges and Woods, 2001). Previous studies aimed at reducing the early morning surge in order to obtain better control of blood pressure and thereby potentially reduce the incidence of ischemic events in these individuals (Patel et al., 2008). This treatment approach, however, did not produce an effective blood pressure control in a significant number of individuals which ended up being subjected to the ischemic events anyway. This led to a newer hypothesis that it was the “dipping status” of the night-time blood pressure that would be more effective in determining the risk of an individual toward an ischemic event i.e., subjects in which the blood pressure dipped at night (“dippers”) were at a lower risk of an ischemic episode in contrast to individuals whose blood pressures did not dip (“non-dippers”) or actually increased (“risers”) (White, 2007). This finding then became the new “gold-standard” for treatment of blood pressure; to cause the blood pressure to dip at night in the “non-dippers” and “risers” in order to achieve blood pressure control and thereby reduce the risk of an ischemic event in these populations.

This led to the development of chronopharmaceuticals that have in several clinical trials proven to be more efficacious in blood pressure management than the previous long-sustained practices (Bridges and Woods, 2001; White and LaRocca, 2002; Sewlall et al., 2010). However, majority of these clinical trials are based on empirical findings and as such lack the fundamental principles on which even better treatment approaches can be built. This is where basic science research is essential in order to determine the molecular mechanisms underlying the pathological onset, maintenance and progression of the disease. Only by clearly defining the disease will it become possible to target molecular culprits and achieve the best possible treatment regimen for subjects at an affordable price and simultaneously reduce the burden on the healthcare system.

## Author contributions

Sumedha W. Karmarkar and Shelley A. Tischkau wrote the manuscript.

### Conflict of interest statement

The authors declare that the research was conducted in the absence of any commercial or financial relationships that could be construed as a potential conflict of interest.
